# Identification of key differentially expressed immune related genes in patients with persistent atrial fibrillation: an integrated bioinformation analysis

**DOI:** 10.1186/s12872-024-04007-6

**Published:** 2024-07-08

**Authors:** Yijing Tao, Tonghui Feng, Lucien Zhou, Leng Han

**Affiliations:** 1 Department of Cardiology, Changshu Hospital Affiliated to Soochow University, Changshu No. 1 People’s Hospital, Changshu, 215500 China; 2 Department of Anesthesia Surgery, Zhejiang Hospital, Hangzhou, 310000 China; 3 Independent researcher, Changshu, 215500 China

**Keywords:** Atrial fibrillation, Inflammatory response, Differentially expressed gene, Biomarkers

## Abstract

**Objective:**

We aimed to investigate key differentially expressed immune related genes in persistent atrial fibrillation.

**Methods:**

Gene expression profiles were downloaded from Gene Expression Omnibus (GEO) using “*GEO query*” package. “*limma*” package and “*sva*” package were used to conduct normalization and eliminate batch effects, respectively. We screened out differentially expressed genes (DEGs) based on “*limma*” package with the standard of |log fold change (FC)| ≥ 1.5 and false discovery rate (FDR) < 0.05. Gene Ontology (GO) and Kyoto Encyclopedia of Genes and Genomes (KEGG) enrichment analyses of DEGs were performed by “*clusterProfler*” package. We further applied LASSO to select key DEGs, and intersected key DEGs with immune related genes from ImmPort database. The ROC curve of each DEIRG was constructed to evaluate its diagnostic efficiency for AF.

**Results:**

A total of 103 DEGs we were screened out, of them, 48 genes were down-regulated and 55 genes were up-regulated. Result of functional enrichment analysis show that, most of DEGs were related to immune response, inflammation, and oxidative stress. Ultimately, *CYBB*, *RORB*, *S100A12*, and *CHGB* were determined as key DEIRGs, each of which displayed a favor efficiency for diagnosing persistent AF.

**Conclusion:**

*CYBB*, *RORB*, *S100A12*, and *CHGB* were identified as key DEIRGs in persistent AF, and future studies are needed to further explore the underlying roles of *CYBB*, *RORB*, *S100A12*, and *CHGB* in persistent AF.

## Introduction

Atrial fibrillation (AF), one of the most prevalent types of arrhythmias, is a leading cause of several adverse clinical outcomes, including stroke, systemic embolism (SE), and heart failure, and is highly related to increased rate of hospitalization and mortality in patients with AF [1]. Given AF is a polygenic and multifactorial disease whose etiologies remains largely unknown, a comprehensive knowledge of the underlying mechanisms of AF is of utmost importance [2].

In the past, significant progress has been made in the treatment of atrial fibrillation (AF). However, there are still many challenges in its management. Current approaches primarily focus on restoring and maintaining normal heart rhythm and preventing thrombus formation. These treatments require high patient compliance, and anticoagulation therapy poses risks of bleeding. Interventional therapies have made substantial advancements in recent years, yet their recurrence rates remain high. There are still many deficiencies in AF treatment, necessitating further exploration of the molecular biology mechanisms underlying AF development to discover more effective therapeutic strategies.

Previous evidence has demonstrated that immune response and inflammation are critical contributors to pathogenesis and progression of AF. A significant increase in serum concentrations of inflammatory-related indicators such as hypersensitive C-reactive protein (hs-CRP), interleukin-6 (IL-6), and tumor necrosis factor-α (TNF-α) was observed in AF patients, with the degree of such increase being proportional to the prognosis of these patients and being capable of predicting the recurrence rate of AF following catheter ablation surgery [3]. Immune cell infiltration, namely, the process in which circulating immune cells migrate to organs/tissues from blood vessels and subsequently give rise to alternations in the local cell microenvironment via releasing multiple pro-inflammatory factors, was initially described as a unique phenomenon accompanying tumor carcinogenesis and metastasis. Later, with the dissection of its diverse role in more non-cancer diseases, this topic started to attract extensive attention worldwide and gradually evolved into a research hotspot in recent years. The extent of inflammatory CD3 + T cells infiltration was markedly higher in the atrial tissues of patients with paroxysmal or persistent AF rather than those of individuals with sinus rhythm (SR), implying that immune cell infiltration may serve as a critical predisposing factor of AF [4]; nevertheless, how to identify high-risk populations of AF through evaluating the status of immune cells infiltration in the atrium remains a formidable challenge and requires to be resolved by further studies. In this study, we retrieved, downloaded, and jointly analyzed the gene expression data of atrial tissues from persistent AF patients from Gene Expression Omnibus (GEO) database.

## Methods

### Data source

GEO is a large database established by National Center of Biotechnology Information (NCBI) that mainly focuses on gathering and classifying gene sequencing data derived from multiple institutions worldwide, and whose aim is to provide an online respository of gene expression profiles, thus serving as a crucial data source for bioinformatics research. All of the GEO data is accessible to the public and can be downloaded freely through: http://www.ncbi.nlm.nih.gov/geo/ [5]. We retrieved all gene expression data of atrial samples from AF patients, including GSE41177, GSE79768, and GSE115574, and downloaded all these datasets using “GEO query” package in R software (version 4.1.3, https://www.r-project.org/) [6–10]. Detailed information about the characteristics of included datasets are illustrated item by item in Table 1.

**Table 1:** The detailed characteristics of the gene chip datasets included in this study

GEO ID	Platform	Reference	Country	SR	AF
GSE41177	GPL570; Affymetrix Human Genome U133 Plus 2.0 Array	Yeh YH, et al. Heart Rhythm, 2013;10(3):383 − 91. PMID: 23,183,193	Taiwan, China	3	16
GSE79768	GPL570; Affymetrix Human Genome U133 Plus 2.0 Array	Tsai FC, et al. Int J Cardiol, 2016;222:104–112. PMID: 27,494,721	Taiwan, China	6	7
GSE115774	GPL570; Affymetrix Human Genome U133 Plus 2.0 Array	Deniz GC, et al. Cardiovascular Therapeutics, 2021, 2021:5516185. PMID: 34,737,791	Ankara, Turkey	15	14

SR, sinus rhythm; AF, atrial fibrillation

### Data filtration

After obtaining all the raw data, we converted the probe expression matrices into gene expression according to the platform annotation files, and performed data normalization with “*limma*” package (https://www.bioconductor.org/packages/release/bioc/html/limma.html). The “*sva*” R package (https://www.bioconductor.org/packages/release/bioc/html/sva.html) was used to eliminate batch effects when integrating different datasets.

### Identification of DEGs and related functional enrichment analysis

The “*limma*” package was also applied to screen for DEGs between AF patients and healthy individuals. The threshold for identifying a DEG included: (1) |log fold change (FC)| ≥ 1.5; (2) false discovery rate (FDR) < 0.05. The results of upregulated or downregulated DEGs were visualized in volcano plots and heat maps constructed by *“ggplot2*” R package (https://bioconductor.org/packages/release/bioc/html/ggplot2.html) and “*pheatmap*” R package (https://bioconductor.org/packages/release/bioc/html/pheatmap.html), respectively [11]. The “*clusterProfler*” R package (https://bioconductor.org/packages/release/bioc/html/clusterProfler.html) was subsequently adopted to implement Gene Ontology (GO) and Kyoto Encyclopedia of Genes and Genomes (KEGG) enrichment analyses of DEGs, with the adjusted *p*-value cutoffs being set to 0.05.

### Identification of key DEIRGs

We screened out key DEIRGs through matching these key DEGs screened out by LASSO with 1788 immune-related genes (IRGs) with known effects on driving immune and inflammatory responses in the ImmPort database [12].

### Analysis of diagnostic efficacy of DEIRGs

The receiver operating characteristic (ROC) curves of identified DEIRGs were created using the “*pROC*” R package (https://www.bioconductor.org/packages/release/bioc/html/pROC.html), and the area under the ROC curve (AUC) was calculated to assess the diagnostic value for AF of each DEIRG.

### Statistical analysis

R software version 4.1.3 (The R Foundation, Vienna, Austria) was used for all bioinformatics analyses, and a two-tailed *P*-value < 0.05 was considered statistically significant.

## Results

### Identification of DEGs

**Fig. 1:**
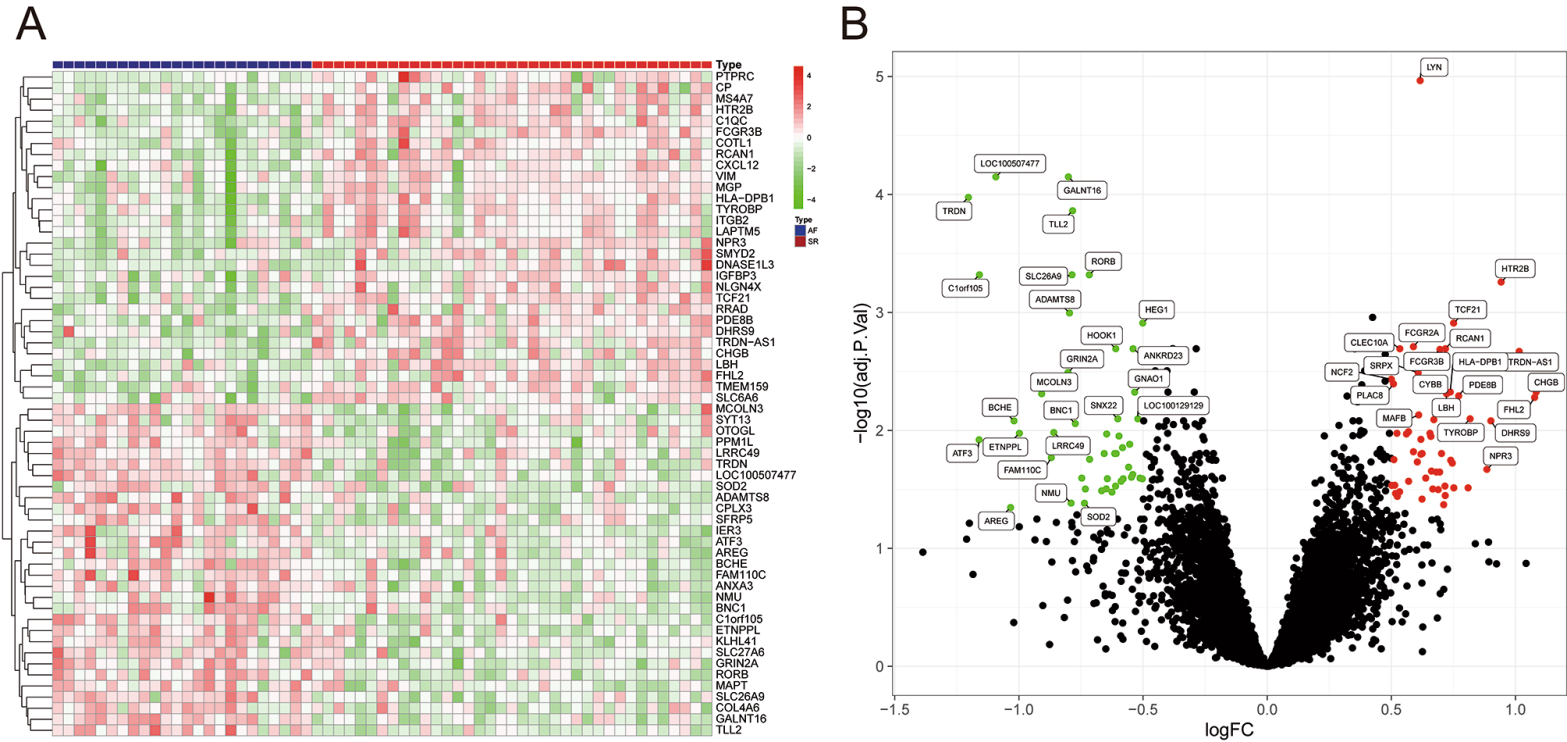
104 DEGs identified in persistent AF. **(A)** Heatmap used to visualize the 104 DEGs; **(B)** Volcano plot used to visualize the 104 DEGs. AF: atrial fibrillation; SR: Sinus rhythm; DEGs: differentially expressed genes

For visualizing the differences in gene expression profiles between persistent AF patients and healthy controls, we constructed a heat map of all 103 DEGs, in which the color of each DEG varies from blue to red depending on its expression levels in different samples (green color for lower expression in AF patients, and red color for higher expression in AF patients) (Fig. 1A), and a volcano plot presenting all DEGs as separate nodes, in which green nodes represented downregulated genes in AF group, red nodes represented upregulated genes in AF group, and the other nodes representing genes with no significant difference in expression levels between two groups were colored black (Fig. 1B).

### Functional enrichment analyses of DEGs

**Fig. 2:**
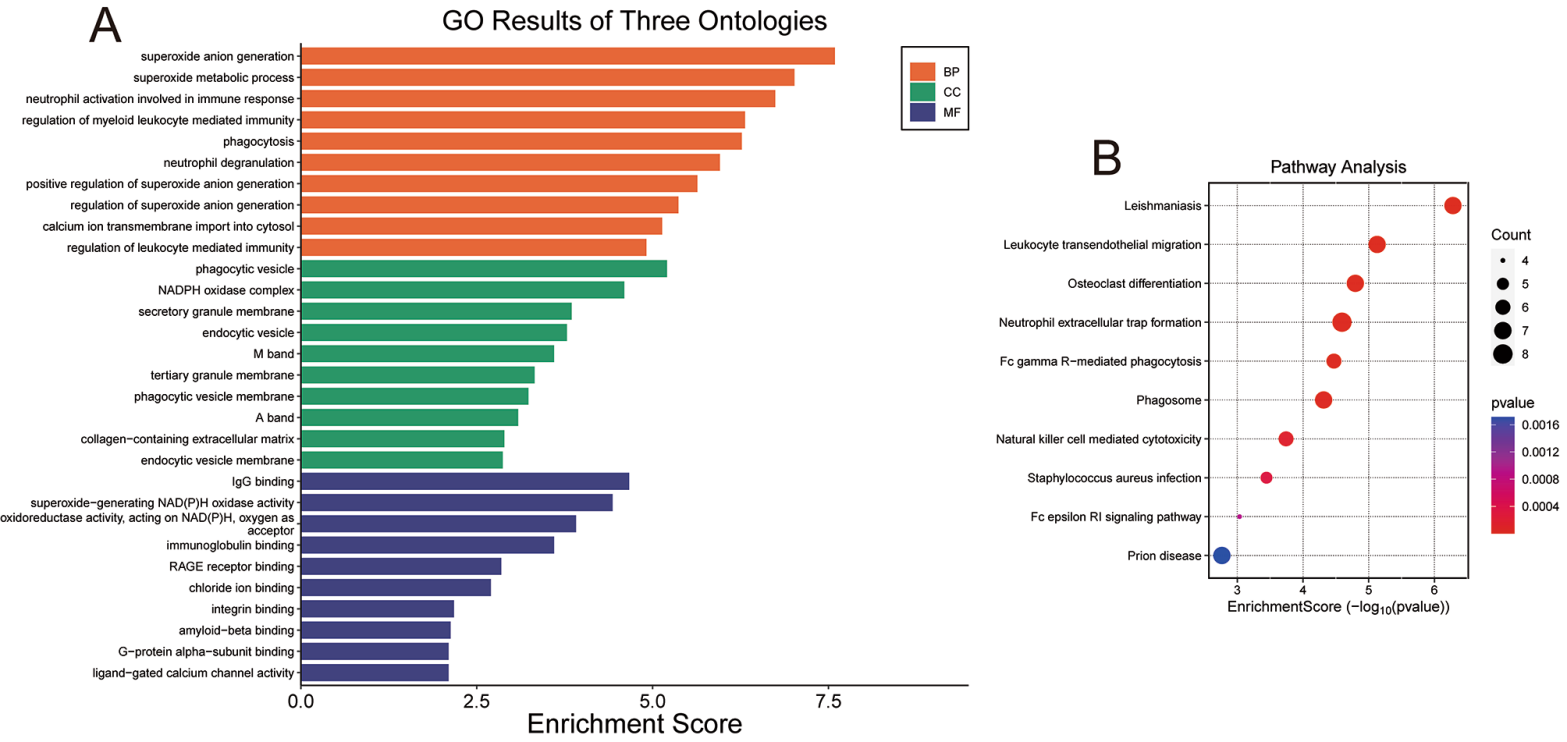
Functional enrichment analysis. **(A)** GO enrichment analysis of DEGs in persistent AF; **(B)** KEGG enrichment analysis of DEGs in persistent AF. AF: atrial fibrillation; BP, biological processes; CC: cell component; MF: Molecular function; DEGs: differentially expressed genes

The biological functions of DEGs were inferred via GO analysis, which focuses on three main aspects, including biological process (BP), cellular component (CC), and molecular function (MF). As shown in Fig. 2A, DEGs was predominantly enriched in BP such as neutrophil activation involved in superoxide anion generation, neutrophil activation involved in immune response, and regulation of myeloid leukocyte mediated immunity, and etc. CC linked with DEGs primarily included phagocytic vesicle, NADPH oxidase complex, and secretory granule membrane, and etc. While major MF linked with DEGs were IgG binding, superoxide − generating NAD(P)H oxidase activity, and oxidoreductase activity, acting on NAD(P)H, oxygen as acceptor, and etc. KEGG analysis revealed that a total of 10 signaling pathways were significantly enriched, including pathways related to Leishmaniasis, Leukocyte transendothelial migration, and Osteoclast differentiation, and etc. (Fig. 2B).

### Identification of key DEIRGs

**Fig. 3:**
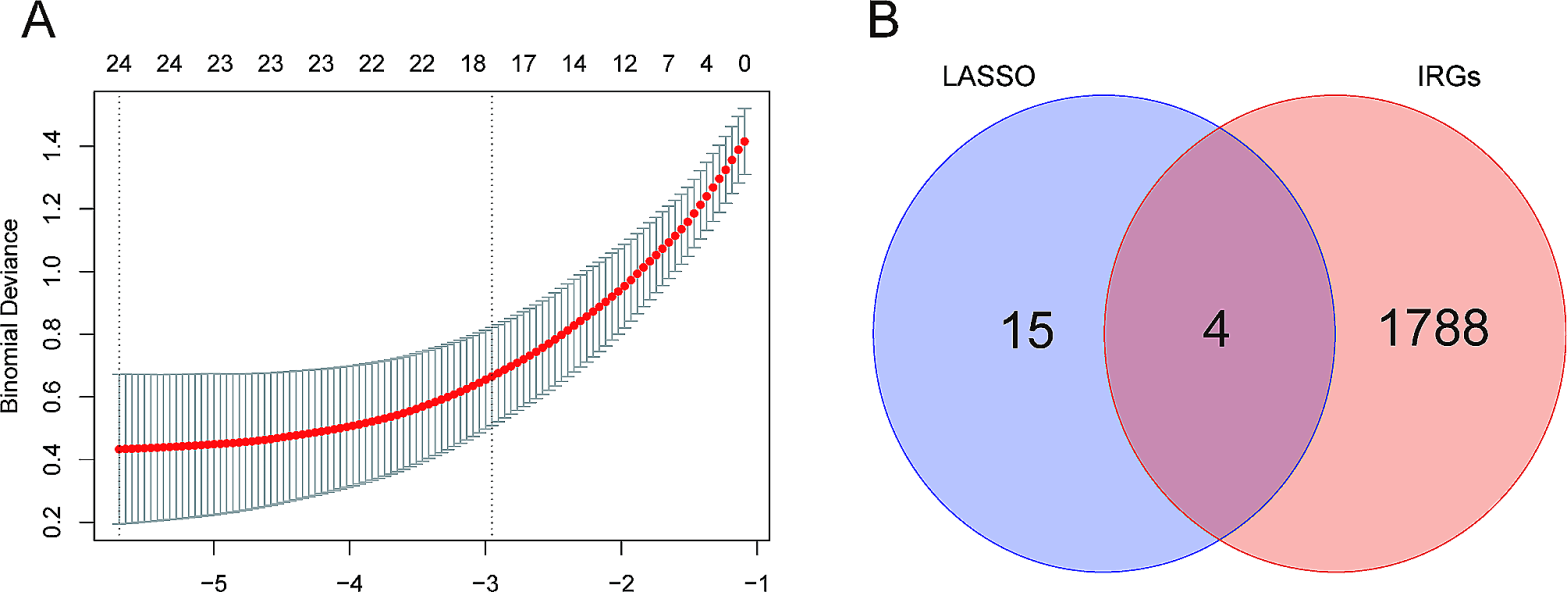
Screening key DEIRGs in persistent AF by LASSO. **(A)** Screening key DEGs in persistent AF by LASSO; **(B)** Venn plot by intersecting key DEGs screened by LASSO and IRGs in ImmPort database. AF: atrial fibrillation; IRGs: Immune-related genes; LASSO: Least absolute shrinkage and selection operator

**Fig. 4:**
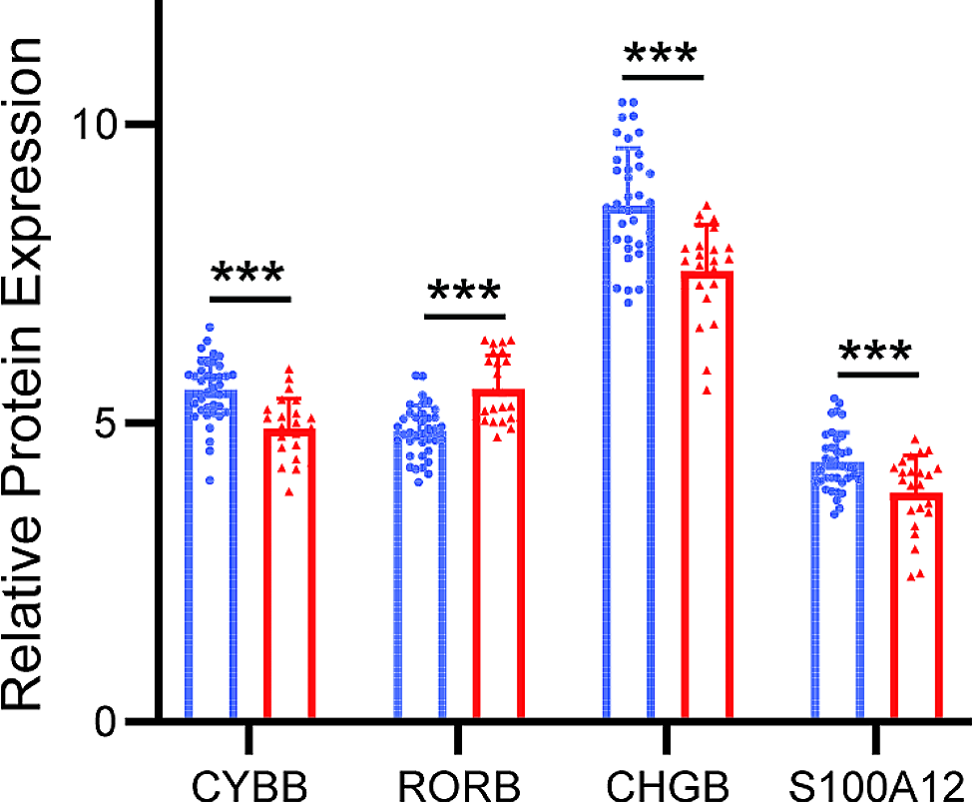
Expression of key DEIRGs among individuals with SR and AF. AF: atrial fibrillation; SR: Sinus rhythm; IRGs: Immune-related genes

Based on LASSO regression algorithm, we extracted 15 DEGs with the strongest relations with persistent AF as key feature genes (Fig. 3A). By matching these DEGs with 1788 IRGs known to have a role in immune and inflammatory response according to the ImmPort database, *CYBB*, *RORB*, *S100A12*, and *CHGB* were further selected as key DEIRGs for persistent AF (Fig. 3B). Among them, the expression levels of *CYBB, CHGB*, and *S100A12* in atrial tissues of persistent AF patients was significantly downregulated, while that of *RORB* was remarkably upregulated (Fig. 4).

### Diagnostic efficacy of key DEIRGs

**Fig. 5:**
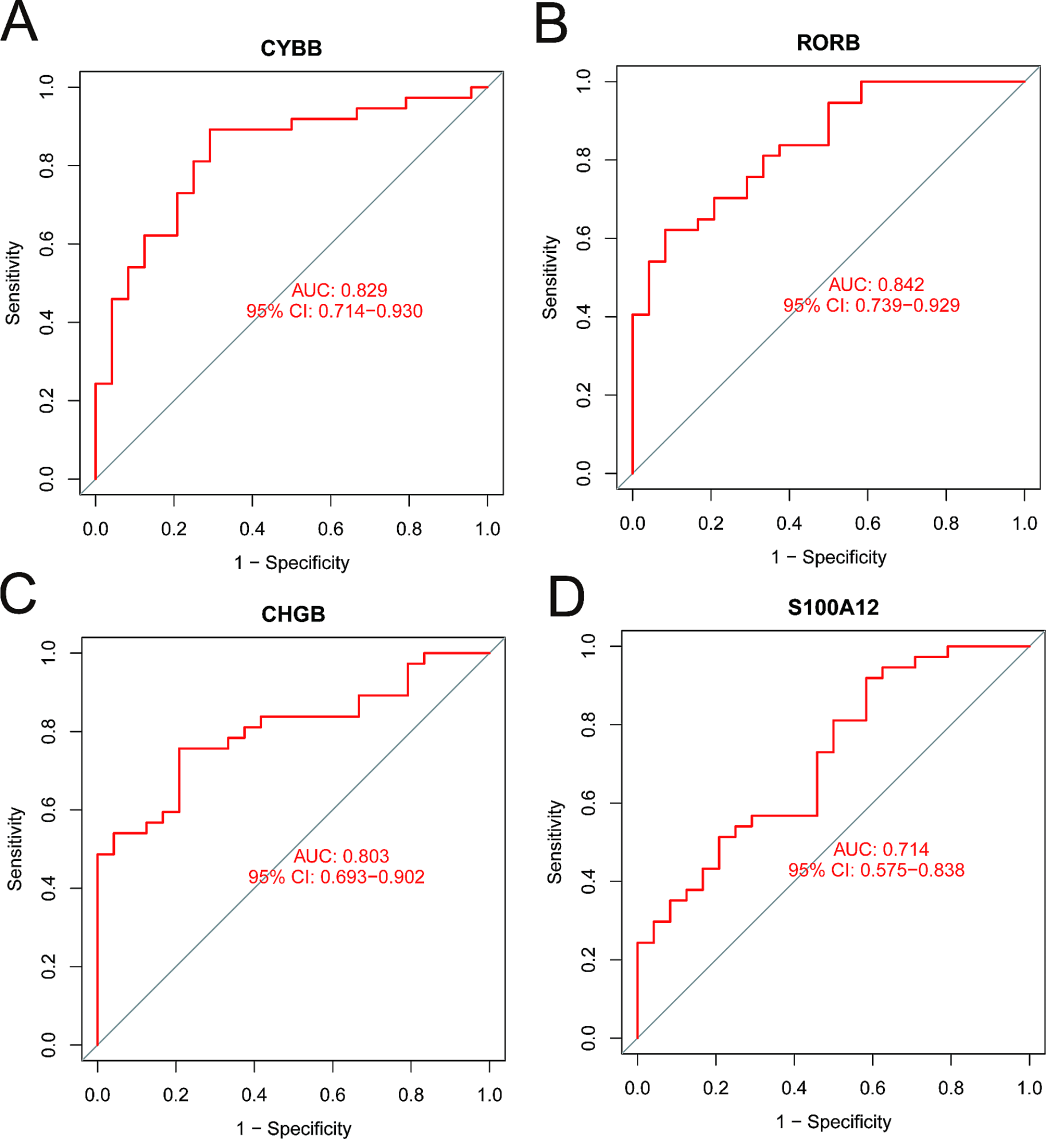
Diagnostic value of key DEIRGs for persistent AF. AF: atrial fibrillation; AUC: area under the ROC curve; IRGs: Immune-related genes

To test whether key DEIRGs can serve as candidate biomarkers for AF, we generated the ROC curves and assessed the diagnostic effectiveness of each DEIRG for distinguishing AF from normal samples (Fig. 5A-D). The AUC of *CYBB, RORB, CHGB*, and S100A12 were 0.829 (95% CI: 0.714–0.930), 0.842 (95% CI: 0.739–0.929), 0.803 (95% CI: 0.693–0.903), and 0.714 (95% CI: 0.575–0.838), respectively, which implied that all these identified DEIRGs had considerable diagnostic efficiency for persistent AF.

## Discussion

In this study, we jointly analyzed the gene expression data of persistent AF patients obtained from GEO database and further determined several DEIRGs related to AF as candidate biomarkers. The primary findings of this study were as follows: (1) compared to healthy individuals, significant changes occurred in the gene expression profiles of persistent AF patients; (2) *CYBB*, *RORB*, *S100A12*, and *CHGB* may represent novel biomarkers for persistent AF; (3) *CYBB*, *RORB*, *S100A12*, and *CHGB* have favor predictive value for persistent AF.

Based on the development of gene sequencing technology, GEO database is increasingly utilized as a powerful tool for providing important data sources for analyzing gene expression profiles specific to different types of arrhythmic disorders, especially AF, which may further facilitate the dissection of underlying mechanisms [5]. We retrieved three datasets of persistent AF generated by different research teams and integrated all the sequencing data of the subjects into a larger dataset in the current analysis, in which factors such as geographic location and ethnic origin were fully taken into account to assess AF-related changes in gene expression profiles more comprehensively [6–10]. It is noteworthy that sequencing platforms of all included datasets are GPL-570, ensuring low heterogeneity among datasets; however, considering the potential differences in experimental conditions among different laboratories, we attempted to assess and adjust the batch effect to guarantee the uniformity of different datasets. In consistent with previous studies, the significance thresholds were set at |log FC| > 0.5 and *P* value < 0.05 when analyzing DEGs [13, 14]. We screened a total of 103 DEGs, which constituted the majority of differences in gene expression profiles between AF patients and normal individuals. Moreover, functional enrichment analyses showed these DEGs are components primarily involved in inflammation and multiple immune-related biological processes.

During recent years, the relationship between inflammation and AF has become a hotspot. Yao et al. found excessively activated NLRP3 inflammasome and related downstream signaling pathways in atrial cardiomyocytes of patients with AF, which can promote ectopic activity, abnormal sarcoplasmic reticulum Ca^2+^ release, atrial effective refractory period shortening, atrial hypertrophy, and ultimately give rise to AF. Both adeno-associated virus-mediated knockdown and genetic deletion of NLRP3 can suppress the development of AF [15]. Lipopolysaccharide (LPS) is a reagent that can induce systemic inflammatory response, and is widely applied in constructing animal models of sepsis. According to recent evidence, the incidence of AF was significantly higher in LPS-treated rats compared to vehicle-treated controls, with the pivotal role of sepsis-induced ferroptosis being implicated in promoting atrial remodeling and AF development [16]. Besides, a retrospective study implied that hs-CRP, a highly sensitive inflammatory biomarker, can be used to predict recurrence rates of AF after catheter ablation surgery [17]. These findings support that immune cells modulate the myocardial microenvironment and interact with neighboring cardiomyocytes via secreting multiple pro-inflammatory mediators, and eventually impair the normal electrical and structural properties of cardiac tissues [18].

Machine learning is representative of an important part of AI and serves as an indispensable tool in bioinformatics research, in which computers are designated to understand how data are structured and organized in a database and then identify and extract key elements from the whole. Contemporarily, machine learning-based algorithms have been successfully applied to assisting in the diagnosis and treatment of multiple diseases [19, 20]. We employed the classical LASSO regression algorithm to determine key DEGs based on gene expression profile data of AF patients. Based on previously published literature, the ImmPort database recorded a total of 1788 IRGs known to play a role in immune and inflammatory responses [21, 22]. All key DEGs were matched with these IRGs, and 4 DEIRGs (*CYBB*, *RORB*, *S100A12*, and *CHGB*) were obtained. We also evaluated the diagnostic performance of all selected DEIRGs and ensured that each DEIRG exhibit considerable diagnostic performance for AF (AUC > 0.75). Among these genes, *CYBB* encodes NAPDH oxidase 2 (NOX2), which is key enzymes catalyzing the production of reactive oxygen species (ROS). Under normal circumstances, ROS participates in various important biological processes, however, excessive synthesis of ROS will lead to pathological conditions such as inflammation [23]. According to a previous study, the expression of NOX2 and NOX4 was dramatically upregulated in diabetic cardiac tissues, which promoted atrial structural remodeling through producing excessive reactive oxygen free radicals and ultimately led to AF [24], which may in turn promote the upregulation of NOX4 expression through inflammation to a larger extent, and eventually form a vicious circle between inflammation and oxidative stress [25]. *RORB* encodes RAR-related orphan receptor B (RORB), a receptor protein known to play an important role in bone metabolism, regulation of circadian rhythm, and other physiological processes, and mutation or abnormal expression of this gene is strongly associated with epilepsy [26]. Despite some studies have proposed that *RORB* can regulate the rhythm of immune T cells and immune system, the exact function of *RORB* in regulating immune cell infiltration and atrial inflammation in AF patients remains poorly understood. *CHGB* encodes chromogranin B, a key protein in the process of catalyzing the formation of catecholamine storage vesicles and regulating sympathetic activity, and thus becomes one of the potentially pathogenic genes related to hypertension [10]. Despite the fact that abnormal *CHGB* function can also lead to oxidative stress, its explicit role in the regulation of immune cell infiltration in atrial tissue in patients with AF remains to be deeply excavated [27, 28].

First, we integrated information from multiple gene sets, which provides a more comprehensive reflection of the changes in gene expression profiles in atrial fibrillation patients. Second, for the first time, we analyzed the gene expression profile changes in persistent atrial fibrillation, which significantly differs from previous studies. However, we have to admit that our study has several shortcomings that need to be noted and addressed in further research: (1) Given details concerning the clinical features of samples in online databases are lacking, it is a tough task to eradicate the potential bias caused by heterogeneities in patient populations and their clinical characteristics. Thus, caution should be taken during the analysis and interpretation of the data; (2) the biological samples used in this study were obtained from both the left and right atria. Different parts of the heart can lead to variations in gene expression profiles; (3) the sample size included in the present study was relatively small, which may pose a great challenge to ensure the accuracy of established findings. Moreover, atrial tissue samples are challenging to obtain and are typically collected during surgeries for underlying conditions, which may also constitute an unavoidable confounding factor in our study. In other words, future prospective studies with larger sample sizes are warranted to verify our conclusions. In addition, more in vivo and in vitro experimental evidence are needed to uncover the explicit roles of identified key DEIRGs in the development of AF and elucidate the underlying mechanisms.

## Conclusion

Our integrated analysis of gene expression datasets from persistent AF patients revealed that four differentially expressed immune-related genes (*CYBB*, *RORB*, *S100A12*, and *CHGB*) have potential to be novel AF biomarkers. Further research is needed to elucidate the mechanisms connecting these genes to immune cell infiltration for the prevention, early diagnosis, and treatment of AF.

## Data Availability

The datasets presented in this study can be found in online repositories. The names of the repositories and accession numbers can be found in the article/Supplementary Material.
